# The Importance of Breast Adipose Tissue in Breast Cancer

**DOI:** 10.3390/ijms21165760

**Published:** 2020-08-11

**Authors:** Charu Kothari, Caroline Diorio, Francine Durocher

**Affiliations:** 1 Department of Molecular Medicine, Faculty of Medicine, Laval University, Quebec, QC G1T 1C2, Canada; charu.kothari.1@ulaval.ca; 2 Cancer Research Centre, CHU de Quebec Research Centre, Quebec, QC G1V 4G2, Canada; Caroline.Diorio@crchudequebec.ulaval.ca; 3 Department of Preventive and Social Medicine, Faculty of Medicine, Laval University, Quebec, QC G1T 1C2, Canada

**Keywords:** breast development, breast cancer, breast adipose tissue, risk factors, therapeutic intervention

## Abstract

Adipose tissue is a complex endocrine organ, with a role in obesity and cancer. Adipose tissue is generally linked to excessive body fat, and it is well known that the female breast is rich in adipose tissue. Hence, one can wonder: what is the role of adipose tissue in the breast and why is it required? Adipose tissue as an organ consists of adipocytes, an extracellular matrix (ECM) and immune cells, with a significant role in the dynamics of breast changes throughout the life span of a female breast from puberty, pregnancy, lactation and involution. In this review, we will discuss the importance of breast adipose tissue in breast development and its involvement in breast changes happening during pregnancy, lactation and involution. We will focus on understanding the biology of breast adipose tissue, with an overview on its involvement in the various steps of breast cancer development and progression. The interaction between the breast adipose tissue surrounding cancer cells and vice-versa modifies the tumor microenvironment in favor of cancer. Understanding this mutual interaction and the role of breast adipose tissue in the tumor microenvironment could potentially raise the possibility of overcoming breast adipose tissue mediated resistance to therapies and finding novel candidates to target breast cancer.

## 1. Introduction

Breast cancer (BC) is the second leading cause of cancer-associated death among women worldwide—constituting 25% of all cancers and 12% of cancer-associated deaths [1]. The breast is mainly composed of adipose and fibroglandular tissues. Breast lobules and ducts are laid upon the background of fibrous and adipose tissues. The breast adipose tissue covers most of the breast from the collarbone to the underarm and around the center of the ribcage [2]. It plays a major role in the communication of all components of the breast microenvironment [3]. The main function of breast adipose tissue is to store the excess energy and release it when required by the body. However, breast adipose tissue also plays a major role in breast development and maturation. Being a rich energy source, it also aids the development and progression of BC. Breast adipose tissue secretes several growth factors which are utilized by cancer cells for their survival. BC typically starts in the epithelial cells surrounding the ductal and lobular tissues of the breast. It then develops a complex microenvironment, which involves all the surrounding cells of the breast including adipose tissue. In this review, we will discuss the importance of the breast adipose tissue ”organ” in breast development through puberty, pregnancy and lactation, up to age-related involution. Thereafter, we will discuss the importance of breast adipose tissue as a ”master regulator” of several processes in the physiology of breast cells, shedding light on the different roles of breast adipose tissue in BC development and progression. Finally, the role of breast adipose tissue in determining the efficacy of BC treatments currently used in clinics will be discussed.

## 2. Adipose Tissue and Its Plasticity

The adipose organ, a dynamic tissue complex and an endocrine organ, consists of adipocytes, a stromal–vascular fraction consisting of lymphocytes, macrophages, endothelial cells, fibroblasts, pericytes, extracellular matrix and adipose precursor cells [4]. It is composed of three different adipose tissues: white, brown and beige. The white adipose tissue (WAT) is the main energy storage compartment consisting of a large cytoplasmic lipid droplet. It releases energy between meals. It is also known to produce many pro-inflammatory molecules, many adipokines related to inflammatory changes and has a low metabolic activity. WAT is characterized by the expression of leptin and S100B and by the lack of uncoupling protein 1 (UCP-1). The brown adipose tissue (BAT) is supposed to be only present in hibernating animals and newborns. However, BAT is also identified in small repositories near the neck and the interscapular region [5]. BAT is characterized by small droplets of lipids, iron-enriched large-spherical and packed mitochondria and a large number of capillaries, which are used for oxygen transport to BAT to produce energy and for the distribution of produced energy to the rest of the body. BAT is responsible for maintaining body temperature by thermogenesis using mitochondria enriched UCP1 protein. Beige/brite (brown-like) adipose tissue is characterized by a role in both energy storage as well as thermogenesis. It expresses UCP-1, PPARγ, leptin, and has a high mitochondrial content compared with WAT [6]. The conversion of WAT to beige/brite adipose tissue has been reported in response to cold or β3-adrenergic agonists [7]. This process is often referred to as browning and can happen after the exposure to the PR-domain containing 16 (PRDM16), fibroblast growth factor (FGF) 21, Peroxisome proliferator-activated receptor-γ (PPAR-γ), PPAR-γ coactivator α (PGCα), irisin, apelin, Cyclooxygenase 2 (Cox2), microRNA 196 (MIR196a) and MIR28 [8]. The conversion of BAT to beige/brite adipose tissue has also been documented. The process is often referred to as the whitening of BAT and is usually considered as BAT malfunctioning leading to death of BAT cells [9]. Furthermore, it has been reported that WAT can trans differentiate to BAT in a cold environment [10,11,12].

## 3. Adipose Tissue in Breast Development

Several studies have demonstrated the role of breast adipose tissue in the morphogenesis of mammary glands. Breast adipose tissue is a major endocrine system of the breast and secretes many growth factors and enzymes. It has been shown by in vitro experiments that breast adipose tissue plays a role in mammary epithelial cell differentiation [13,14]. Experiments using a co-transplantation system with breast stromal cells have shown that breast adipose tissue is responsible for characteristic morphogenesis of epithelial cells in the breast [15]. Further, it has been shown that loss of WAT of breast—using A-ZIP/F1 mouse model—results in reduced fertility and distends mammary ducts [16]. A study by Hu et al. 2002 showed the complete loss of ductal epithelium development when inherited loss of functional leptin occurs or in the absence of the leptin receptor, whereas the structure of ductal epithelium is restored with the re-establishment of leptin signaling [17]. On the other hand, abnormal mammary growth with underdeveloped ducts is observed in the presence of overexpression of adiponectin in mice [18,19,20]. Leptin and adiponectin are secreted by breast adipose tissue in the breast.

**Figure 1:**
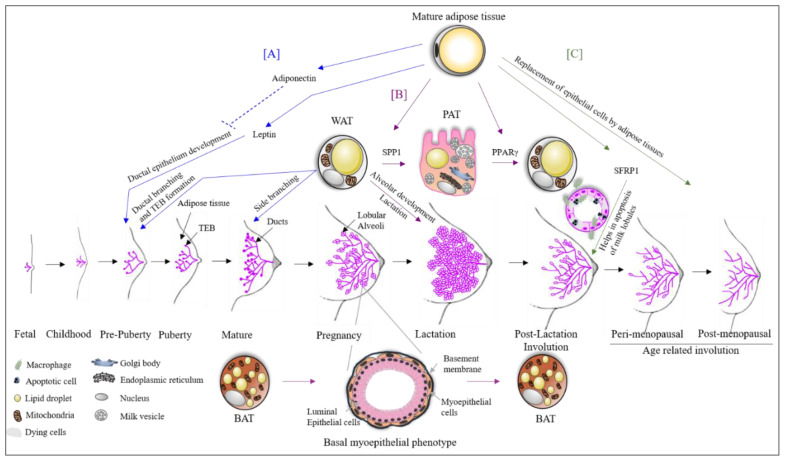
The role of adipose tissue in breast development. (**A**) Mature breast adipose tissue secretes leptin which is essential for ductal epithelial development. High levels of adiponectin inhibit this process. White adipose tissue (WAT) is essential for the formation of terminal end buds (TEBs) during prepuberty and puberty stages. After menarche, the breast starts to mature, and the duct starts its side branching, which requires WAT. (**B**) During pregnancy, WAT trans differentiates into pink adipose tissue (PAT). PAT has milk secretory potential. The process of trans differentiation from WAT to PAT is carried out by the transcription factor SPP1. Moreover, WAT is also essential for alveolar development during the lactation phase and also for the lactation process. During the phase of pregnancy and lactation, brown adipose tissue (BAT) trans differentiates to a basal myoepithelial phenotype helping in the alveolar development. Both trans differentiated cells revert to their original state after lactation with the aid of the transcription factor PPARγ. (**C**) There are two stages of breast involution (i) post-lactation and (ii) age-related. SFRP1 secreted by breast adipose tissue helps in these involution processes by the apoptosis of luminal epithelial cells. Furthermore, the epithelial cells are replaced by adipose tissue during the involution process.

Landskroner-Eiger et al., 2010 showed that breast adipocytes play a crucial role in mammary gland development during prepuberty, puberty and adulthood. It is known that during the prepuberty to puberty phase, rapid ductal branching and terminal end bud (TEB) formation take place, whereas during adulthood, alveolar buds start to develop side branching (Figure 1) [21]. This study also highlights that the loss of mammary gland specific adipocytes results in a slowdown of all these processes, leading to fewer duct branching points, fewer TEBs, excessive lobulation and changes in proliferation and apoptosis of TEBs associated with epithelium, pointing towards the importance of breast adipose tissue in the development as well as the maintenance of the breast [21].

## 4. Adipose Tissue Plasticity during Pregnancy, Lactation and Involution

The trans differentiation of adipose tissue underlines the extraordinary property of adipose tissue plasticity and is the result of physiological changes [22,23]. A recent study shows a broader view of this picture in pregnancy [24]. WAT in the breast has been shown to change during the second stage of pregnancy into milk producing glands containing lipid-enriched elements, an apical surface with microvilli and fully developed rough endoplasmic reticulum (Figure 1). The milk producing adipose glands are parenchymal cells of the breast adipose organ [24] and are named Pink adipose tissue (PAT). PAT arises exclusively in female subcutaneous depots during pregnancy and lactation. It is so-called this because during pregnancy, the mammary gland looks pink at the macroscopic level. The existence of PAT in the breast is also confirmed by the presence of the whey acidic protein (*WAP*) gene, a marker of the milk-producing epithelial mammary gland only present in pregnant women adipose tissue [25]. Perilipin 2 (Plin2) is also expressed in PAT, but is not present in WAT [26,27]. PAT does not express Plin1, which is typical of WAT [28]. The trans differentiation of WAT to PAT during pregnancy and lactation has been confirmed by the expression of these two genes (*Plin1* and *Plin2*) on day 17–18 of pregnancy in compartmentalized adipocytes or early alveoli from the mammary gland section of mice [26]. The expression of Plin1 decreases as pregnancy progresses. Another study shows that 10–15% of early post-lactation adipocytes showed the expression of both WAP and S-100B confirming the PAT to WAT differentiation post-lactation [29]. A study by Couldrey et al., 2002, showed that the absence of WAT in the breast results in the inhibition of alveolar development and lactation in mice (Figure 1) [7]. Prokesch et al., 2014, showed that secreted phosphoprotein 1 (SPP1) signaling through integrin plays a role in WAT to PAT trans differentiation [26]. On the other hand, it has been shown that PPARγ is a key factor for PAT to WAT trans differentiation in the breast, which occurs post-lactation during involution (Figure 1) [24]. Another study reported that the downregulation of PPARγ expression results in a pro-breast tumorigenic environment [30], suggesting that PAT to WAT trans differentiation is an essential step in proper involution. Furthermore, it has been shown that partial involution directly results in the malignant transformation of normal breast cells [31]. In addition to partial post-lactation involution [32,33,34], an increase in breast adipose tissue (replacing the epithelial cells of glands) [35,36] results in excessive pro-inflammatory mediators, leading to tumorigenesis. The importance of breast adipose tissue in proper breast involution was also indicated in the study on Secreted Frizzled Related Protein 1 (SFRP1). SFRP1 is a known adipokine secreted by adipocytes, and it plays a role in adipogenesis, inflammation and apoptosis [37,38,39] processes that are all of significant importance in breast involution (Figure 1). Furthermore, SFRP1 overexpression was observed in the post-lactation period [40] indicating its role in involution. Gauger et al., 2012, also suggested that SFRP1 knockout mice show a ductal and lobular branching similar to mid-pregnancy mice [41]. This further indicates an important role of breast adipose tissue in breast involution and tumor development.

The role of BAT has also been studied in pregnancy. It was shown that BAT in the breast changes to a mammary basal myoepithelial phenotype during pregnancy and lactation (Figure 1) [42]. The lineage tracing experiments identified the expression of a gene signature resembling BAT and myoepithelial cells in 2.5% of the anterior dorsal interscapular mammary myoepithelial cell population. When traced during the involution process after lactation, 0.8% of BAT was trans differentiated from myoepithelial cells [42]. A study by Singh et al., 2017, reported a role for BAT and beige/brite adipose tissue in BC development [43]. Markers for BAT (UCP1, MYF5, EVA1 and OPLAH) and beige (UCP1, CD137/TNFRSF9 and TBX1) adipocytes were significantly high in BC xenografts [43]. 

These findings further strengthen the role of breast adipose tissue throughout the development of the mammary gland and is an essential factor in pregnancy, lactation and involution processes. A dysregulation in the proper functioning of the mammary adipose tissue has an adverse effect in breast development, leading to tumorigenesis. Furthermore, the multiplicity of breast adipose tissue subtypes and its association with breast further highlights the importance of personalized BC care.

## 5. Transcriptional Regulation of Adipose Tissue

Transcriptional regulation of gene expression is a key factor in adipogenesis and has an important role in breast development and BC (Table 1).

Adipogenesis is the term used for the formation of new fat cells. The most important transcription factor that has a role in adipogenesis is PPARγ. PPARγ is often regarded as the master regulator of adipogenesis as its expression is significantly induced during adipogenesis. The loss of function of PPARγ due to mutation can lead to lipodystrophy, insulin resistance and diabetes in humans [88,89,90]. Wang et al., 2013, showed that in mice, adipocyte-specific deletion of PPARγ results in the complete loss of WAT [44]. Another important transcription factor in adipogenesis is CCAAT-enhancer-binding proteins (C/EBP) α. Both PPARγ and C/EBPα regulate each other in a positive feedback loop, leading to an increase in adipogenesis genes. C/EBPα expression is induced around 2–4 days after adipogenesis, showing a difference from other C/EBPs which mostly work at a pre-adipogenesis stage. Furthermore, C/EBPα knockout mice show a reduced expression of BAT and a loss of WAT [91]. There are several other transcription factors affecting adipogenesis: C/EBPβ, C/EBPδ, CHOP, EBF1/2, SREBP1, KLFs, GATA2/3, PREF-1, SIRT-1, TAZ, Wnt, FoxA2 and FoxC2, all having a role in pre-adipogenesis, and they mediate their effect by altering the expression of PPARγ and c/EBPα [92]. Whereas C/EBPβ, C/EBPδ, EBF1/2, SREBP1 and KLF3/4/5/6/15 promote adipogenesis [93,94], GATA2/3, CHOP, FoxA2, FoxC2, Wnt signaling, PREF-1, SIRT-1, TAZ and KLF2/7 have a role in anti-adipogenesis [93,94]. At the pre-adipogenesis stage, C/EBPβ is considered to be the most important transcription factor. After binding to the adipogenic enhancer sites on PPARγ and C/EBPα, it assists other adipogenic transcription factors such as the glucocorticoid receptor, STAT5A and RXR 9 to form adipogenic enhancers [49]. PPARγ and C/EBPα both play a role in the terminal differentiation of adipogenesis and are both considered tumor suppressors [45,46,47,48,95]. However, evidence suggests that PPARγ does not initiate tumor formation in normal breasts whereas in a tumor environment, the expression of PPARγ results in tumor progression signaling [45]. Other evidence shows a downregulation of PPARγ due to the activation of the Wnt signaling pathway in many cancers [95].

In terms of function, most pre-adipogenesis regulators in BC are involved in tumor growth and proliferation. c/EBPβ has an important role in breast development. C/EBPβ−/− mice showed delayed ductal outgrowth, ductal ectasia, decreased branching, reduced secretory activity, decreased levels of milk protein β-casein and WAP [50,51]. An increase in C/EBPβ mRNA is associated with estrogen receptor (ER) negative BC [52,53,54,55]. A significant association is observed with metastatic BC [56], high grade tumor [55,57,58] and poor survival outcome [56]. Furthermore, Balamurugan et al. 2019, showed that a downregulation of C/EBPδ leads to a reduction in stemness of BC cells which is mediated by linking IL-6 and Hif-1α signaling [59]. Studies suggest a tumor suppressive role of Early B Cell Transcription Factor (EBF)s [96]. On the other hand, Sterol regulatory-element binding protein (SREBP) 1 has been shown to be associated with tumor metastasis and poor progression of BC patients [60]. There is a mixed literature regarding a role for the Krüppel-like family of transcription factors (KLF) in BC. It has been reported that KLF5, KLF6-SV, KLF4α and KLF7 have a role in tumor progression, epithelial to mesenchymal transition (EMT) and metastasis [61,62,63], whereas KLF2, KLF6, KLF4 and KLF15 inhibit proliferation, metastasis and cell cycle in BC [62,64].

The adipogenesis repressors also display mixed roles in BC. GATA-binding factor (GATA) 2 promotes BC by inhibiting PTEN activity [65], while GATA3 acts as a tumor suppressor and is required for the normal development of the mammary gland, specifically luminal epithelial cells [66]. Further, Forkhead Box (Fox) A2 suppresses BC [67], whereas FoxC2 promotes BC [68]. CHOP (C/EBPζ) and Wnt signaling suppress adipogenesis by promoting the differentiation of mesenchymal stem cells into myocytes and osteocytes but blocking the commitment to the adipocyte lineage [69,70]. CHOP correlates with the invasiveness of human colorectal cancer [71], but not much information is reported in BC. Wnt signaling (dependent or independent of CTNNB1) is required for the development of the mammary gland, its branching and functions [72,73,74,75,76,77]. It has been reported that high levels of Catenin Beta 1 (CTNNB1) lead to high tumor grade and poor prognosis in BC patients [78,79,80]. Moreover, Preadipocyte factor 1 (PREF-1), also known as DLK-1, is highly expressed in mesenchymal adipocyte precursors, which are important for the development of embryonic WAT and the expansion of adult adipose tissue [81,82]. During breast development, platelet-derived growth factor (PDGF) receptor α+ (PDGFRα+) and PREF-1+ mesenchymal stem cells, located near the parenchymal epithelium, can differentiate into adipocytes or epithelial cells depending on the stimuli from steroid hormones [83]. PREF-1 has been shown to exert its effect in a dose-dependent manner in BC, where high levels of PREF-1 result in a decrease in cell proliferation and invasion, whereas a low-level expression is necessary for these processes [84]. The role of Sirtuin 1 (SIRT-1) in BC is controversial. Latifkar et a.l, 2019 showed that a knockdown of SIRT-1 changes the secretome of BC cells, leading to increased invasiveness and survival [85]. On the other hand, Jin et al., 2018, showed that SIRT-1 expression leads to tumor promotion by modulating the expression of AKT [86]. Tafazzin (TAZ) is highly expressed in most aggressive BCs and has a role in BC migration, invasion and tumorigenesis [87].

The regulators of breast adipose tissue have been extensively studied, depicting the importance of adipose tissue in puberty, breast development, pregnancy and involution. There is a delicate switch which balances the outcome of this important organ—breast adipose tissue as a regulator of important functions, or as a tumor promoter. 

## 6. Adipose Tissue as a Master Regulator

**Figure 2:**
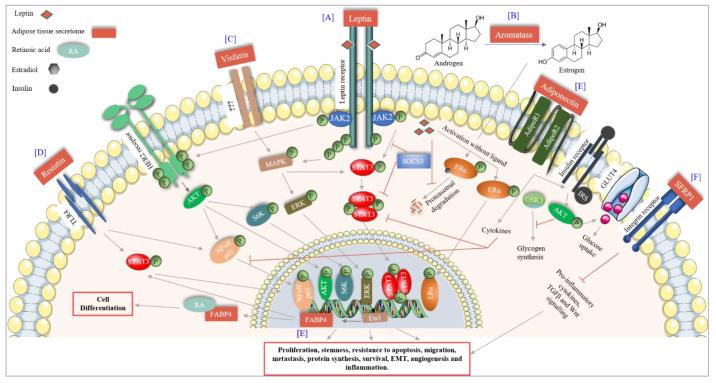
Adipose tissue signaling in breast cancer. (**A**) Leptin, a major adipocytokine released by adipose tissue binds to the leptin receptor and activates the JAK2-STAT3 pathway leading to proliferation and stemness of BC cells. Leptin transactivated HER2 receptor in absence of its ligand via JAK2, which leads to BC survival. Leptin also activates the ER receptor in absence of estrogens and, furthermore, inhibits its proteasomal degradation. (**B**) Aromatase secreted by adipose tissue converts androgens to estrogens leading to an increase in BC risk. (**C**) Visfatin increases the MAPK-ERK pathway through an unknown receptor thereby increasing BC proliferation. Activated MAPK also activates STAT3 signaling. (**D**) FABP4 expression is transcriptionally activated by the ERK-Ets1 pathway. Upon activation, FABP4 increases proliferation and migration of BC cells by nuclear accumulation of PPARγ and SREB2. Furthermore, FABP4 translocates to the cytoplasm where it binds to RA and, therefore, leads to cell differentiation. (**E**) Adiponectin increases the insulin signaling, glucose uptake, decreases glycogen synthesis and inhibits STAT3 and NFκB mediated signaling leading to the inhibition of BC survival and progression. (**F**) SFRP1 signaling through the integrin receptor inhibits pro-inflammatory cytokines, TGFβ and Wnt signaling, thereby inhibiting BC progression.

The breast adipose tissue is a known endocrine organ that secretes many factors including adipokines, cytokines, chemokines and growth factors, therefore controlling various cellular processes. Factors with a particular significance in BC are discussed in this section (Figure 2).

### 6.1. Adipocytokines 

Adipokines or adipocytokines are cytokines secreted by adipose tissue (both pre- and mature adipocytes) with autocrine and paracrine functions. There are several adipokines secreted primarily by adipocytes, namely Leptin, Adiponectin, Resistin and Chemerin.

#### 6.1.1. Leptin

Leptin is a predominantly adipocyte-specific peptide hormone, encoding a 16kDa protein. It has a dual role as a hormone and a cytokine. It regulates food intake and energy homeostasis leading to fat degradation in adipocytes. A role for leptin has been reported in puberty, the estrous cycle and pregnancy. High levels of leptin in obese females result in early menarche [97]. The estrous cycle in females has been associated with high levels of energy flux and energy balance. If body fat is extremely low, it affects leptin levels and in turn the menstruation cycle resulting in poor egg quality [98], which underlines the importance of leptin in women’s physiology. However, an increasing amount of data suggests a role for leptin in female cancers including breast, cervical, endometrial and ovarian [99]. The effect of leptin expression and its association with BC differs between premenopausal and postmenopausal women. In postmenopausal women, high amounts of leptin have been associated with BC, whereas in premenopausal women, it is supposed to reduce the risk of BC. In premenopausal women, leptin has a role in folliculogenesis of the ovary, and high levels of leptin show a protective role against BC by decreasing the levels of estradiol [100,101]. Leptin mediates its role by binding to the leptin receptor (obRa-obRf), a class 1 cytokine receptor present in breast cells and overexpressed in cancer. Leptin activates multiple downstream signaling via SOCS-STAT3 transcription activation, thereby activating MAPK and AKT pathways, resulting in increased proliferation, angiogenesis and decreased apoptotic death in cancer cells [102]. It can also activate ER (in the absence of the ligand) [103,104] and human epidermal growth factor receptor 2 (HER2, by transactivation of EFGR and JAK2), therefore resulting in tumor progression and resistance to targeted therapies [105,106].

#### 6.1.2. Adiponectin

Adiponectin is an adipocyte-derived protein hormone, with a molecular weight of approximately 30 kDa (244-amino acid, encoded by the *ADIPOQ* gene), and circulates in the plasma as a low (trimer), medium (hexamer) and high (multimer) molecular weight protein. It is also secreted from the placenta during pregnancy [107]. It increases glucose uptake (decreases glucogenesis), fatty acid oxidation, insulin signaling (by downregulating mTOR pathway), eNOS activity vasodilation and decreases inflammation (by inhibiting IKK-NFĸB-PTEN signaling) [108]. Adiponectin mediates its effect by binding to adiponectin receptors (AdipoR1 and AdipoR2) as well as T-cadherin. In BC, adiponectin has been reported to have antiproliferative and proapoptotic effects. Adiponectin activates the cytokine signaling responsible for the inactivation of leptin-induced AKT and STAT3 activation as well as Wnt signaling [109,110]. It is suggested that low levels of adiponectin are associated with high grade tumors, which might be due to an interference in its signaling via AMPK, which has an antiproliferative effect [111]. Studies also suggest that considering leptin or adiponectin concentration individually in plasma is not correct, the true measurement is the ratio of the two, as different concentrations of each can be found in BC cell lines [112,113,114,115]. Studies indicate that high ratios of leptin to adiponectin increase the risk of BC in postmenopausal women [113,114], and also the progression of triple-negative BC (TNBC) [115].

#### 6.1.3. Resistin

Resistin is another adipose tissue-specific peptide hormone also known as adipose tissue-specific secretory factor (ADSF) or C/EBP-epsilon-regulated myeloid-specifically secreted cysteine-rich protein (XCP1). It is a 12.5kDa cysteine rich protein (108 amino acids), which causes an increase in low density lipoproteins (LDL) and is found in inflammatory zones. It is also secreted by immune cells (monocytes, macrophages and bone marrow cells) [116,117] and is known to increase inflammation, resistance to insulin and atherosclerosis [118]. Resistin has been shown to have inverse effects in various cancers including BC. It is responsible for BC progression and increased stemness properties, which are primarily mediated by TLR-4 leading to the induction of NF-κB and STAT3 pathways, two important pathways in cancer progression [119]. It also induces autophagy in BC cells leading to the resistance of doxorubicin-induced apoptosis [120]. 

Chemerin is a 14kDa protein also known as retinoic acid receptor responder protein 2 (RARRES2), tazarotene-induced gene 2 protein (TIG2) or RAR-responsive protein TIG2. It is mainly secreted by WAT as a pro-chemerin and is cleaved by inflammatory or coagulation serine proteases to its active form [121]. It functions as a leukocyte chemoattractant by binding to chemokine-like receptor 1 (CMKLR1). It has a role both in adaptive and innate immunity. It is an adipokine that has a function in adipogenesis as well as adipocyte metabolism [122]. There are conflicts concerning its role in BC. A study by Pachynski et al., 2019, suggests that it inhibits BC by recruiting immune effector cells in the tumor microenvironment [123]. On the contrary, a study by El-Sagheer (2018) shows a correlation of high chemerin expression with the poor survival outcome of BC patients [124]. Being a chemoattractant, it can attract natural killer cells and dendritic cells, thus behaving as a tumor suppressor [125]. On the contrary, it can also induce inflammation and increase angiogenesis [126], which explains the contradictory behavior of this protein in BC and, therefore, needs to be thoroughly analyzed in different contexts.

### 6.2. Other Cytokines Secreted by Adipose Tissue

#### 6.2.1. Lipocalin 2

Lipocalin 2 is an approximately 23kDa secretary glycoprotein also known as oncogene 24p3 or neutrophil gelatinase-associated lipocalin (NGAL). It was identified in neutrophils, but a recent study suggests that it is also secreted by adipocytes [127]. Its main function is in the activation of innate immune response. It also acts as a transporter for small hydrophobic molecules. In BC, lipocalin 2 promotes cancer progression by increasing EMT [128]. Further clinicopathological analyses reveal that the localization of lipocalin 2 also plays an important role in BC outcome. High cytoplasmic and low nuclear localization of lipocalin 2 was associated with the worst survival outcome in BC patients [129]. 

#### 6.2.2. Visfatin

Visfatin is a 52kDa protein, also known as nicotinamide phosphoribosyltransferase (NAmPRTase or Nampt) and pre-B-cell colony-enhancing factor 1 (PBEF1). It is expressed by many tissues and by adipocyte tissue macrophages. Visfatin induces BC proliferation via ERK1/2 and AKT pathways [130] and induces a malignant potential in BC by c-Abl and STAT3 activation [131]. A role for visfatin has also been seen in BC stemness by upregulating the growth differentiation factor 15 (GDF15) and by the activation of the AKT pathway [132].

#### 6.2.3. Plasminogen Activator Inhibitor 1 (PAI-1)

Plasminogen Activator Inhibitor 1 is a serine protease inhibitor also known as the endothelial plasminogen activator inhibitor or serpin E1. It is secreted by various cells including ECs, megakaryocytes, smooth muscle cells, fibroblasts, monocytes, adipocytes, hepatocytes and other cell types. Its main function is to inhibit tissue/urokinase plasminogen activator (tPA/uPA) inducing fibrinolysis [133]. However, it also plays a role in adhesion, migration, signal transduction and anti-apoptosis [134,135]. In BC, a high expression of PAI-1 has been associated with shorter disease-free survival [136]. Furthermore, it has been shown that the expression of PAI-1 leads to resistance to the Src inhibitor via an increase in the secretion of CCL5 in HER2-positive BC cells [137].

#### 6.2.4. Fatty Acid Binding Protein 4 (FABP4)

Fatty Acid Binding Protein 4 is a 15kDa protein, also known as adipocyte protein 2 (aP2). It is predominantly expressed in adipocytes, macrophages and dendritic cells [138]. Adipocytes release FABP4 during lipolysis via nonclassical pathways. FABP4 is an intracellular chaperon involved in the trafficking of lipids [139]. It constitutes 1% of all secreted proteins from adipocytes [140]. A study by Guaita-Esteruelas et al., 2017, showed that the exogenous expression of FABP4 leads to BC progression by the activation of MAPK and AKT pathways in combination with the activation of fatty acid transport proteins [138]. Another study by Apaya et al., 2020, showed that the expression of FABP4 in combination with CYP2C19 and FABP5 is required in both metastasis and stromal interactions of TNBC. It further shows that in presence of metabolic by-product epoxyeicosatrienoic acid, FABP4 translocate to the nucleus resulting in the nuclear accumulation of PPARγ and SREBP-2, resulting in increased proliferation, migration, transformation and distant metastasis of TNBC cells [141].

#### 6.2.5. Interleukins

Interleukins are a group of cytokines first observed in leukocytes. Interleukins are believed to modulate immune response and inflammation. Adipocytes secrete many interleukins (ILs) such as IL-1, IL-1β, IL-6, IL-8 and IL-10. IL-1 has a role in both innate and adaptive immunity, where it mediates the inflammatory response in the presence of different stimuli [142]. In BC, IL-1 and IL-1β have been reported to induce tumorigenesis and bone metastasis by regulating the tumor microenvironment. In normal breast biopsy, IL-1 is not detected but is significantly increased in BC along with IL-2, IL-4, IL-10 and G-CSF [143]. The increase in IL-1 expression in BC is usually considered as poor progression, since the expression of IL-1 induces a secondary response by activating the secretion of other inflammatory molecules (cytokines, chemokines), and also by altering the expression of adhesion molecules that have a predominant role in metastasis [144]. In a tumor microenvironment, IL-1 is mainly secreted by adipose tissue. It has a role in stimulating the secondary response; therefore, even a very small amount of IL-1 secretion is considered as a most alarming adipokine response in BC [145]. On the other hand, IL-6 is considered to be a pleiotropic cytokine, with contrasting effects on BC cells, both protective and inhibitory [146]. However, recent evidence shows that adipocyte derived IL-6 has a role in BC proliferation, EMT, stemness, angiogenesis, cachexia and resistance [147,148,149,150]. A similar finding has been observed with IL-8 secreted by adipocytes in BC, where IL-8 secreted by cancer-associated adipocytes is 2-fold higher than in normal cells. It plays a role in BC growth, progression and also in the increase in other tumor promoting factors [151]. Furthermore, Vazquez Rodriguez et al., 2018, showed that breast adipocytes in ER+ BC induce the primary stem cell dissemination leading to metastasis via the secretion of IL-8, which enhances a pro-inflammatory and pro-tumorigenic environment necessary for metastasis [152]. Moreover, secreted IL-8 can induce osteoclastogenesis and bone resorption in the case of bone metastasis of BC cells [153]. IL-10, like IL-6, also has both a protective and an oncogenic role in BC. Ahmad et al., 2018, showed that both IL-6 and IL-10 expression correlate with a better survival outcome in early stages of invasive BC [154], whereas other data suggest that the expression of IL-10 is responsible for BC cell evasion from apoptosis [155]. IL-10 is also the immunosuppressive cytokine released by BC to evade immune response [156].

#### 6.2.6. Tumor Necrosis Factor (TNF)

TNFα, another cytokine secreted by adipose tissue and macrophages, plays a role in the acute phase reaction and in many signaling pathways resulting in activation, differentiation, survival and cell death, and this also holds true in BC. TNFα increases tumorigenesis primarily by altering the expression of matrix metalloproteases (MMPs) and dipeptidylpeptidases [157,158]. Many studies report the involvement of TNFα in fibroglandular tissue augmentation [159,160] and BC progression [158,161,162]; however, TNFα expression also has a role in the normal regulation of adipose tissue. It has been shown that TNF-α plays a physiological role in premenopausal (non-obese) women by regulating adipogenesis or lipid storage in adipocytes, thereby establishing the total volume of adipose tissue [163,164]. 

### 6.3. Chemokines

The adipose tissue secretes many chemokines. Among them are CXCL2, CXCL5, CXCL8, CXCL10, SDF-1, MCP-1 (CCL2) and MIP-1α (CCL3). Chemokines are cytokines with a chemotactic behavior. They are widely studied in BC and are usually associated with inflammation and tumorigenesis [165,166,167]. Zhao et al., 2018, showed that CCL5 secreted by adipose tissue derived stem cells (ADSC) increases BC proliferation [168]. Furthermore, a study by Picon-Ruiz et al., 2016, shows that BC cells grown in coculture with immature adipocytes (ADSC differentiated to adipocyte lineage) display a cytokine profile enriched in IL-6, IL-8, CCL2 and CCL5. These cytokines result in increase in BC cells tumorigenesis and metastatic potential [148]. Cytokines are not only secreted by the stroma (consisting of adipose tissue) but also by BC cells themselves [143,169]. For instance, the normal epithelial cells of the breast do not display the expression of MCP-1, whereas its expression is very high in BC cells. This two-way secretion of chemokines increases the inflammatory response in the tumor-microenvironment, where adipose tissue plays the most significant role. Chemokine secretion in BC is responsible for increased invasiveness and metastasis [170]. 

### 6.4. Growth Factors

Adipose tissue secretes many growth factors including vascular endothelial growth factor (VEGF), hepatocyte growth factor (HGF), nerve growth factor (NGF), insulin growth factor (IGF) and PDGF [171,172]. Growth factors are well studied in nearly all cancers including BC. The signaling mediated by the above-mentioned growth factors has been discovered in BC progression, survival, angiogenesis, invasion and metastasis [173,174]. Furthermore, the crosstalk between growth factors is known in BC as well as other cancers, and this leads to resistance to therapies [175,176,177,178].

### 6.5. Other Proteins, Aromatase, Fatty Acids and Cholesterol

Adipocytes express many other proteins such as osteopontin (OPN), SFRP1, Allograft inflammatory factor 1 (AIF1) and collagene. OPN, also known as SPP1, was first identified in osteoblasts. It plays a role in matrix remodeling, calcification and as a chemokine to induce immune response [179]. A recent report highlighted its expression in adipocytes, and a study identified that the expression of SPP1 was significantly higher in the adipocytes near the BC site as compared with adipocytes from normal breast tissue [180]. Our group has shown that SPP1 is associated with invasiveness of BC and that its expression in BC increases with the progression of the disease [181]. Furthermore, it also regulates the function of adipocytes by altering its differentiation and by increasing its inflammatory signaling by inducing the expression of the integrin, CD44 and inflammatory cytokines [182,183].

SFRP1 is an adipokine mainly expressed in mature adipocytes with a role in adipogenesis [38]. SFRP1 mediates its effect on adipogenesis in a paracrine manner by inhibiting the Wnt/β-catenin pathway, thereby determining the fate of ADSC to become an adipose tissue [38]. Its expression has been correlated with mild obesity but significantly decreases with morbid obesity. Furthermore, it has been shown that SFRP1 expression decreases the expression of IL-6, MCP-1 and adiponectin, thereby decreasing the pro-inflammatory response of adipose tissue in BC [39]. Klopocki et al., 2004, showed that the loss of SFRP1 leads to poor prognosis in early stage BC [184]. Another study by Gregory et al., 2017, identified a role for SFRP1 in regulating the transcription factor Early Growth Response 2 (EGR2) via the TGFβ pathway [185]; the loss of SFRP1 leads to BC progression by upregulating TGFβ and thereby EGR2 [185].

A study from our group showed that breast adipose tissue also expresses AIF1 [186]. AIF1 is a new human adipokine implicated in adipose inflammation in obese women [187] and produced by macrophages within human WAT [188]. Our study identified two isoforms of AIF1 expression in breast adipose tissue, namely AIF1 splice variant 1 (AIF1v1) and AIF1v3. The expression of AIF1 was significantly correlated with the infiltration of lymphocytes in BC tissue, suggesting a role for AIF1 in the tumor microenvironment [186].

The adipose tissue also consists of an extracellular matrix (ECM) which embeds adipocytes. The ECM of breast adipose tissue is enriched in Collagen VI [189]. Collagen VI has a role in inflammation, angiogenesis and EMT [190]. 

The breast adipose tissue also secretes estrogen synthetase, also called aromatase, which synthesizes estrogen from androgen. It has been well established that the risk of BC is higher in women with longer exposure to estrogens [191]. In premenopausal women, the ovary is the main organ producing estrogens [192]. However, in postmenopausal women, as the ovary becomes nonfunctional for estrogen production, the adipose tissue becomes the main source of estrogen [193], therefore, extending the exposure of the body to hormones rendering postmenopausal women more prone to BC. The cytokines secreted by the adipose tissue itself, namely TNFα and IL-6, can increase the production of aromatase either in an autocrine or a paracrine manner, thereby increasing the production of estrogen [194]. Moreover, leptin secreted by adipose tissue has been shown to induce the transcription of ER in BC cells, independent of estradiol [104]. Leptin further increases the synthesis of the estrogen-inducible protein pS2, which increases the nuclear localization of ER [195]. These data suggest that the adipose tissue secretome in postmenopausal women leads to both an increase in ER and its ligand, resulting in the extended exposure of breast epithelial cells to estrogen hormone, thereby increasing the risk of BC. In addition, hormonal replacement therapy is often offered to postmenopausal women to provide relief from menopausal symptoms without considering that this therapy might increase the risk of BC.

Breast adipose tissue is also a source of free fatty acids and cholesterol required as a constant source of ATP by cancer cells to meet their requirement of increased proliferation and energy requirements [196]. 

In summary, it is apparent that breast adipose tissue secretion affects different aspects of BC by affecting tumor progression, angiogenesis, metastasis and the tumor microenvironment. Additionally, it alters the expression profile of BC cells. As described by Fletcher et al., 2017, when BC cells are grown in conditioned media from adipose tissue explants from BC patients, there is an increase in the expression of versican, CD44, ADAMTS1 and adipoR1 in BC cells resulting in increased proliferation, adhesion and migration. On the other hand, conditioned media from adipose tissue explants from normal breast cells decrease the migration of BC cells [197]. The study by Fletcher et al., 2017, further highlights that the natural secretome of breast adipose tissue is not always tumor promoting. The changes in the secretome profile of breast adipose tissue could be designated as the crosstalk between breast cells and the adipose tissue. The secretome profile of adipose tissue is also altered in the case of obesity, leading to increased secretion of inflammatory molecules, thereby resulting in BC progression.

## 7. Risk and Prognostic Factors for BC and Involvement of Adipose Tissue

**Figure 3:**
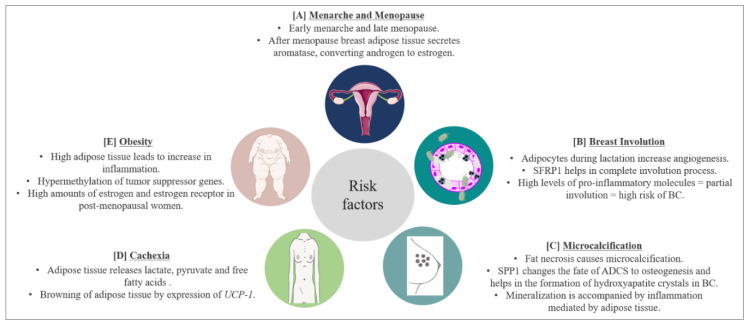
BC risk factors and prognostic factors and the role of adipose tissue. (**A**) Early menarche and late menopause increase the risk of BC by increasing the exposure of breast cells to estrogen hormones. Breast adipose tissue secretes aromatase which converts androgen to estrogen further complicating the scenario. (**B**) Breast adipose tissue secretes SFRP1 which helps in post-lactation involution age-related breast involution. Involution involves the inflammation process leading to apoptosis of epithelial cells. High levels of pro-inflammatory molecules secreted by breast adipose tissue lead to partial involution which increases the risk of BC. (**C**) Microcalcification is a predisposing factor for BC. Microcalcifications due to fat necrosis resemble malignant microcalcifications further complexifying prognosis of BC. Furthermore, SPP1 secreted by breast adipose tissue changes the fate of adipose derived stem cells (ADSC) to osteogenesis, increasing BC risk. Moreover, breast adipose tissue increases the inflammatory process accompanying mineralization. (**D**) During BC progression, BC cells signal lipolysis of surrounding adipose tissue to meet the energy requirement of BC. Adipose tissue releases lactate, pyruvate and free fatty acids. WAT also starts to express UCP-1, thereby trans differentiating to BAT and exhausting the energy source of the body. This leads to cachexia and eventually death. Around 30–50% of cancer-associated deaths are due to cachexia. (**E**) Obesity is a worldwide problem and known to increase BC risk. High adipose tissue increases the inflammatory process and hypermethylation (resulting in inhibition) of tumor suppressor genes. Obesity also increases the expression of estrogen and estrogen receptors in postmenopausal women, enhancing the risk of BC.

There are several risk and prognostic factors associated with BC. In this section, we will discuss some, with respect to adipose tissue involvement (Figure 3).

### 7.1. Menarche and Menopause 

Early menarche has been considered as a risk factor for BC due to the increased exposure of breast cells to estrogens. The role of leptin in early menarche in obese patients has been discussed in the previous sections. Although early menopause means shorter exposure to estrogen, data suggest that after menopause, the adipose tissue becomes the main secretory organ for estrogens. In obese women, this becomes a major problem due to the abundance of adipose tissue [198]. Adipose tissue in the breast produces a high amount of aromatase which converts androgens secreted by the ovary in postmenopausal women [199,200]. Estrogen secreted in such a fashion in the breast increases the concentration of estrogen in the breast by 10 times in comparison with the concentration present in the circulation, thereby increasing the risk of BC [201]. Among BC patients, our group found higher levels of estradiol in breast adipose tissue of women diagnosed with ER-positive BC as compared to those with ER-negative BC [202].

### 7.2. Involution

The breast undergoes two kinds of involution: a post-lactation involution and an age-related lobular involution (around/after menopause). During pregnancy, breast stroma undergo a massive remodeling to make room for new growing epithelial cells. This remodeling sometimes results in pregnancy associated BC (PABC). PABC can occur during pregnancy, lactation, or post-lactation involution. PABC is usually ER and progesterone receptor (PR) negative, is often diagnosed at later stages and predominantly has a worse prognosis [203,204]. Data suggest a significant role of adipose tissue in PABC occurring during lactation or post-lactation involution. McCready et al., 2014, showed that breast adipocytes present during lactation promote tumorigenesis by increased vasculogenesis. This leads to an increase in vascular endothelial cells resulting in increased angiogenesis [205]. Furthermore, it has been shown that leptin can stimulate the growth of ER-negative BCs [206]. Post-lactation involution resembles wound healing, where an extensive immune response takes place. This leads to apoptosis of epithelial cells, which are replaced by stromal cells enriched in adipocytes [207]. The site of involution is enriched with TGFβ, VEGF, TNFα and IL6 [208], which have all been shown to promote cancer, and are also secreted by breast adipose tissue. On the other hand, a review from our group has highlighted that there could be a possible link between the post-lactation overexpression of SFRP1 and complete involution [37], as mice lacking expression of SFRP1 display a breast morphology that is similar to mid-pregnancy mice [41]. This could also suggest a protective role of breast adipose tissue against BC.

Age-related lobular involution (ARLI) is an irreversible change in the breast which is marked by a decrease in the number and size of breast lobules [209]. ARLI leads to a decrease in epithelial cells, which are replaced by stromal cells including adipocytes. The ARLI is inversely correlated with BC risk, as a very small amount of epithelial cells remains to be transformed into malignant cells after involution. On the other hand, no involution or partial involution increases the risk of BC [210,211]. A study from our group has shown that high levels of pro-inflammatory molecules in breast tissue can lead to partial involution and, therefore, could be correlated with high BC risk [212]. The increase in pro-inflammatory molecules could be attributed to the adipose tissue present in the breast. It has been reported that in the breast epithelium of premenopausal women, around 10% of epithelial cells are ER positive; however, 90% of the epithelial cells in postmenopausal women are ER positive [213], which increases the risk of BC in postmenopausal women. After lobular involution, the breast is enriched with adipose tissue, leading to an increased production of estrogen via androgens. This combination of increased ER and its ligand works in favor of BC after menopause and the complexity increases in obesity where there is a further increase in adipose tissue.

### 7.3. Microcalcifications

Microcalcifications in breast are recognized as a possible predisposing factor for BC. Usually, calcification is indicative of a benign process due to some injury or due to calcium deposition from serum. However, certain predisposing factors in microcalcification can indicate the possibility of an emerging cancer smaller in size (< 0.5 mm each). Microcalcifications have varying sizes and shapes, and are often worrying if they are branching, rod-like, or angular and if they are clustered in one area of the breast [214]. One cause of microcalcification is fat necrosis. Microcalcifications due to fat necrosis are pleomorphic, focally clustered and are often undistinguishable from microcalcifications associated with malignancy [215,216], leading to unnecessary intervention in many instances. However, the mineralization process which occurs during microcalcification leading to the production of calcium oxalate or calcium hydroxyapatite is increased by several processes including inflammatory response. The adipose tissue in breast has been reported to mediate acute inflammatory response in BC leading to cancer progression. Furthermore, a microcalcification event can be marked by changes in the transcription factors regulating ADSC, changing their fate to osteogenesis. Studies have shown that breast adipose tissue is an important site for the presence of ADSC [217,218]. Among the transcriptional regulators of ADSC is SPP1, which is also secreted by adipose tissue [219]. It has been shown that SPP1 is responsible for the formation of hydroxyapatite crystals in BC cells in response to an osteogenic cocktail [220]. Furthermore, the expression of SPP1 is significantly elevated in calcified BC [221], and the expression of SPP1 also aids in bone metastasis of BC cells [222]. Once BC cells metastasize to bone cells, they colonize in bone-marrow adipose tissue [223], where SPP1 modulates the ECM component of the bone microenvironment to promote BC progression [224]. Oyama et al., 2002, showed that infiltration of foam cells expressing SPP1 in the breast is responsible for breast microcalcification and atypical cystic lobule formation [222]. Foam cells are macrophages present in fat cells. Studies show that the presence of microcalcifications in BC leads to poor clinical outcome [225,226,227,228]. 

### 7.4. Cachexia

Cachexia is defined as a gradual decrease in the adipose tissue storage by the body and lean body mass. Around 30–50% of cancer-associated deaths are due to cachexia. During this process, metabolic changes occur; cancer cells behave like parasites, taking nutrition from the surrounding tissue leading to exhaustion of the body’s energy resources [229]. Cancer-associated cachexia not only increases the rate of mortality but is also responsible for treatment failure [230,231]. The adipose tissue and the muscle cells play a major role in this process, releasing lactate, pyruvate and free fatty acids upon the signals received by cancer cells [232]. In BC, it has been shown that exosomes released from cancer cells trigger cachexia [233]. Moreover, the adipose tissue adjacent to BC cells starts expressing the UCP-1 gene responsible for the browning of WAT [233], resulting in increased cachexia. Cachexia is also induced by several cytokines such as TNFα, IL-1, IL-6 and IFNγ [234]. A recent review by Rybinska et al., 2020, showed an increase in TNFα, IL-6, IL-1β, CCL2, adiponectin and collagen and a decrease in leptin in BC causing anorexia leading to cachexia. Furthermore, BC mediated cachexia is also associated with a decrease in adipogenesis transcription factors PPARγ, c/EBPα, GLUT4 and SREBP-1, and an increase in HSL, an enzyme responsible for lipolytic activity in cachexia [235]. 

### 7.5. Obesity

Obesity is defined as an excess accumulation of fat in the body, with great health risk to an individual. According to the world health organization, there is a three-fold increase in obesity since 1975 [236]. Obesity has been linked to an increase in the risk of BC and also with poor survival outcome [237,238]. Around 60% of women in the USA are overweight, and research has established a clear link between Body Mass Index (BMI) and BC [239]. However, BMI is not an absolute parameter, as a lean woman could also have a significant depot of visceral fat, which might not be reflected by BMI measurement. Therefore, the current standard followed in clinical settings also includes waist-to-hip ratio. Borugian et al., 2003, showed that waist-to-hip ratio measurement in combination with menopausal and ER status is a predictable marker of BC associated deaths [240]. Moreover, a study by our group suggests that the weight gained during adulthood could be associated with BC risk in women with more fatty breast; therefore, a minimum of breast fat may be needed to promote the development of BC [241]. Besides the known adverse effects of excess adipose tissue in BC, a systemic review from our group highlighted that the obesity linked hypermethylation of *PTPRN2* and *ABLIM2* genes in breast tissue could be associated with BC [242]. 

## 8. Therapeutic Approaches for BC and the Importance of Adipocytes

**Figure 4:**
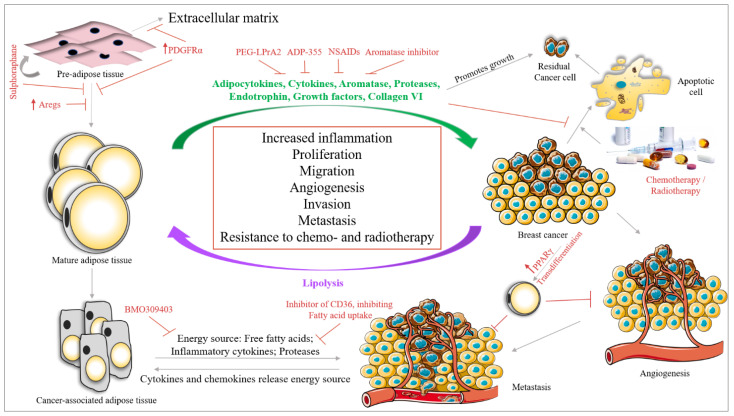
Interaction between BC cells and adipose tissue in the BC microenvironment. Breast adipose tissue secretes various molecules (green) which increase BC survival, proliferation, migration, angiogenesis and metastasis. These secretomes also help BC cells in evading chemotherapy mediated apoptosis and increase the survival of residual cancer cells after chemotherapy. BC cells also secrete cytokines and chemokines which signal lipolysis of adipose tissue, changing the secretory phenotype of adipose tissue to a cancer-associated phenotype. The cancer associated adipose tissue aids in BC survival by releasing inflammatory cytokines, proteases and providing the energy source in the form of free fatty acids. Studies have identified various approaches to target the crosstalk between BC cells and adipose tissue (indicated in red). By targeting the differentiation of pre-adipose tissue to mature adipose tissue, which can be performed by overexpressing adipogenesis regulatory cells (Aregs stop this differentiation), PDGFRα (changes the fate of pre-adipose tissue to extracellular matrix) or by treatment with Sulforaphane (which stimulates the regeneration of pre-adipocytes and inhibits its differentiation to mature adipocytes). Furthermore, the inhibition of fatty acid transporter 4 (BMO309403) inhibits the transfer of energy from adipose tissue to BC cells. Moreover, the inhibition of CD36 by monoclonal antibody against CD36 leads to the inhibition of fatty acid uptake by BC cells. Another study showed that increasing the expression of PPARγ in BC cells could lead to trans differentiation of BC cells into adipose tissue, thereby inhibiting BC angiogenesis.

There are several approaches under trial to deal with different subtypes of BC. In this section, we will focus on a few approaches, with adipose tissue at the center of the discussion (Figure 4).

### 8.1. Targeting Adipose Tissue and BC Cell Crosstalk

Targeting adipose tissue and BC crosstalk can be performed at various levels, by inhibiting (i) adipogenesis, (ii) the secretome of adipose tissue and (iii) the signals given by BC cells modulating the behavior of adipose tissue.

Regarding the first approach, a study by Schwalie et al., 2018, reported the presence of adipogenesis regulatory cells (Aregs) in WAT stromal vascular fraction, characterized by high expression of the cell surface protein CD142 and the ATP-binding cassette sub-family G member 1 (ABCG1). In their study, they have shown that Aregs inhibit the differentiation of adipocyte precursor cells (APC) into adipocytes. Triggering the expression of Aregs in the breast adipose tissue population could be a new therapeutic approach [243]. Furthermore, Miwa et al., 2018, showed that APC expresses the PDGFR tyrosine kinase α (PDGFRα), which is absent in mature WAT [244]. The activation of signaling via PDGFRα in APC represses its differentiation into WAT by converting APC to ECM, which could be a potential target in inhibiting adipogenesis [245]. Moreover, a drug called Sulforaphane has been shown to inhibit adipogenesis by activating the self-renewal process of mesenchymal stem cells in BC [246].

Various approaches are ongoing to target the secretome of adipose tissue. Rene et al., 2009, showed that the leptin peptide receptor antagonist 2 (PEG-LPrA2) shows antitumor activity in ER-positive/negative BC by suppressing VEGF signaling [247]. The adiponectin receptor agonist ADP-355 has been shown to inhibit BC cell growth in in vitro and in vivo systems [248].

As BC is marked by a significant increase in inflammatory molecules both within the tumor and in the microenvironment, anti-inflammatory drugs are being considered with other drugs. Nonsteroidal anti-inflammatory drugs (NSAIDs) such as ibuprofen, mefenamic acid, celecoxib, aspirin and diclofenac mediate their action by inhibiting COX (COX-1 and COX-2). COXs are enzymes that play a role in prostaglandins secretion [249], thereby regulating inflammatory processes such as platelet aggregation. Gao et al., 2007, identified 3 variants in *COX-2* that, when occurring simultaneously, increase the risk of BC in Chinese women [250]. The use of NSAIDs is still under consideration as it could affect the gastrointestinal tract, the platelet function and increase the risk of cardiovascular diseases [251,252]. Anti-inflammatory molecules used in the treatment of HIV infection are also under investigation in BC. For example, maraviroc, vicroviroc and leronlimab targeting CCR5 are under clinical trial for BC [253]. Furthermore, Cenicriviroc for CCL2 [254], Tocilizumab targeting IL-6R [255], Canakinumab for IL-1β [256], Infliximab and nanoparticle based CYT-6091 (under clinical trial) for TNF-α [257,258] can be used as possible anti-inflammatory molecules in BC. 

Another possible approach is to hamper the communication between adipose tissue and cancer cells. A study by Nieman et al., 2011, showed that inhibiting the fatty acid transport from adipose tissue using an inhibitor of the fatty acid binding protein 4, BMS 309403, could inhibit the energy supply needed by cancer cells resulting in the suppression of tumor growth and proliferation in ovarian cancer [259]. Moreover, as the process is bidirectional, inhibiting the transmembrane protein CD36, which is responsible for the uptake of fatty acids in cancer cells could also inhibit tumor growth. Pascual et al., 2017, showed that the neutralizing-antibody against CD36 completely inhibits metastasis in melanoma and BC cells [260]. Moreover, several aromatase inhibitors (AI), namely anastrozole, letrozole and exemestane have been evaluated in clinical trials in postmenopausal women [261]. It has been postulated that AI could have different effects on postmenopausal obese women with BC as compared with postmenopausal non-obese women with BC [262,263]. However, data from our group show that although there is a significant difference in the estrogen levels between postmenopausal obese women and postmenopausal lean women, there is no significant difference in estrogen levels between AI-treated obese and non-obese postmenopausal women with BC [264]. The results from our group, therefore, do not support the argument that ineffective outcome with AI could be the result of obesity measured by BMI, where estrogen secretion from excess adipose tissue compensates for the decrease in estrogen levels achieved by AI.

Furthermore, a recent approach discusses the trans differentiation of BC cells to adipose tissue, thereby inhibiting BC metastasis. EMT is a major phenomenon in metastasis, highlighting the plasticity of the cell. Using this plasticity and changing the fate of the cells by PPARγ, Ishay-Ronen et al. differentiated BC cells to fat cells, thereby inhibiting BC metastasis [265]. 

Adipose tissue interaction with available therapies: adipocytes have been shown to confer resistance to various BC therapies. The breast adipose tissue matrix is enriched in collagen VI which releases endotrophin (a cleavaged product of the collagen VI(α3) chain). Endotrophin induces EMT in BC cells. A study by Park et al., 2013, shows that endotrophin secreted by adipose tissue confers resistance to cisplatin in BC mouse models [266]. Lyes et al., 2019, showed that ADSC secretes FGF2 which activates ERK signaling, thereby promoting the proliferation of chemotherapy residual TNBC cells through the SDF-1α/CXCR4 signaling pathway [267]. Furthermore, Yeh et al., 2017, showed that ADSC promotes doxorubicin resistance in TNBC by CXCL1 secretion, which upregulates the expression of ABCG2, an ATP binding cassette (ABC) transporter [268] responsible for multidrug resistance in cancers [269]. Duong et al., 2015, showed that adipocytes can induce resistance to trastuzumab by interfering with interferon γ secretion by NK cells in HER2-expressing BC cells [150]. Furthermore, adipocytes segment the autotaxin-lysophosphatidic acid signaling which confers resistance to taxol in BC cells by blocking the binding of Taxol to tubulin [270]. Moreover, the adipose tissue adjacent to a growing tumor confers resistance of the tumor to radiotherapy by secreting IL-6, which upregulates Chk1 responsible for a radio-resistance phenotype [271].

### 8.2. Breast Reconstruction

BC is considered among the most devastating diseases in women. Mastectomy at an early stage is considered the most reliable treatment option. Breast reconstruction after surgery is, therefore, often practiced. This is performed by autologous fat grafting using ADSC, with or without enrichment with the stromal vascular fraction (SVF), PDGF and hormones including insulin [272,273]. To enhance the efficiency of grafting, ADSC is used. The use of PDGF enhances the proliferation and differentiation of the graft [274], while SVF increases the angiogenesis in the graft, an important aspect for breast regeneration [275]. However, all these components (ADSC, PDGF and SVF) have the capacity to induce tumorigenesis if any residual tumor cells are left, leading to relapse. Therefore, mixed opinions exist regarding breast reconstruction surgery. However, data so far are not strongly convincing that a reconstruction surgery with ADSC shows more relapse, but the involvement of SVF or PDGF needs to be carefully evaluated [276].

### 8.3. Physical Activity

Physical activity has beneficial outcomes in BC patients. It has been shown that regular exercise can decrease the risk of BC, BC recurrence and also increase the survival outcome of patients [277]. Results from our team indicate that regular physical activity can reduce the local inflammatory profile in the breast [278], high involution in ARLI, low dense breast [279] and the decrease in circulating sex hormones [280], thereby reducing the risk of BC. Furthermore, studies have shown that regular physical activity can reduce pain, fatigue and increase bone quality, physical functioning of BC patients and BC survivors [281,282,283,284,285]. Exercise leads to a decrease in body fat mass. A study by Brown et al., 2016, showed that physical activity reduces the metabolically active, energy-rich adipose tissue and, therefore, the risk of BC [286]. Furthermore, it has been shown that the secretome profile of adipose tissue changes depending upon lifestyle: active or sedentary. Physical activity changes the secretome of adipose tissue, thereby reducing the risk of BC [287].

## 9. Conclusions

There are multiple layers in the biology of adipose tissue. While we know part of it, a large amount is still not understood. The present review highlights the absolute importance of adipose tissue in the normal development of the breast. However, the multilayered complexity of adipose tissue also serves a major role in BC. Furthermore, the interaction between breast adipose tissue and cancer cells in the BC microenvironment is a complex network of both the autocrine and paracrine effects of secretory molecules from both cell types (adipose tissue and cancer cell), modulating each other’s function for a common goal, i.e., BC survival and proliferation. A detailed knowledge of this complexity gives a fair idea about friends (such as PPARγ, c/EBPα, GATA3, KLF2, FoxA2, SFRP1 etc.) and foes (such as c/EBPδ, KLF5, GATA2, FoxC2 and SPP1) in the process of BC and of where and how we could use this information in targeting BC. However, the most important factor is to maintain the balance of nature by maintaining a healthy lifestyle and maintaining energy intake and expenditure, as various studies show the undeniable positive effect of physical functioning on BC survival [277,278,279,280,281,282,283,284,285,286,287].

## 10. Major Teaching Points

1. Adipose tissue is required for the development of the breast throughout a life span of a women (embryonic development, puberty, pregnancy and lactation).
2. Adipose tissue plasticity plays a major role in pregnancy and lactation by converting to PAT and epithelial cells.
3. Correct involution requires proper adipose tissue functioning.
4. Adipose tissue derived from the breast of BC patients shows different secretory profiles compared with those isolated from healthy individuals.
5. Adipose tissue plays a major role in BC risk, progression, migration, metastasis and resistance to available therapies.
6. Targeting the crosstalk between adipose tissue and BC, in combination with known therapies, could be a possibility to overcome obstacles.

## Figures and Tables

**Table 1:** The role of transcription factors in adipogenesis, breast development and breast cancer.

Transcription Factor	Role in Adipogenesis	Role in Normal Breast	Role in Breast Cancer
PPARγ	Terminal differentiation of adipocytes	Absence leads to complete loss of WAT [44]	PPARγ expression act as a tumor suppressor but in a tumor microenvironment helps in tumor progression [45]
c/EBPα	Terminal differentiation of adipocytes	Transcriptional regulation in early stage of lactation and in later involution process [46]	Tumor suppressor [46,47,48]
c/EBPβ	Promotes adipogenesis. Transcription factor at pre-adipogenesis. Assist other adipogenic transcription factor [49]	Helps in ductal outgrowth, ectasia, its branching and secretory activity [50,51]	Associated with ER negative BC, high grade tumor, metastasis and poor survival outcome [50,51,52,53,54,55,56,57,58]
c/EBPδ	Promotes adipogenesis	ND	Increases BC stemness [59]
SREB1	Promotes adipogenesis	ND	Tumor metastasis and poor progression [60]
KLF5/KLF6-SV/KLF4 and KLF7	Promotes adipogenesis	ND	Tumor progression, EMT and metastasis [61,62,63]
KLF2/KLF6/KLF4 and KLF15	Promotes adipogenesis	ND	Inhibits proliferation, metastasis, and cell cycle in BC [62,64]
GATA2	Promotes adipogenesis	ND	Promotes BC by inhibiting PTEN [65]
GATA3	Promotes adipogenesis	Normal development of mammary gland, specifically luminal epithelial cells [66]	Tumor suppressor [66]
FoxA2	Inhibits adipogenesis in pre-adipocytes and increases glucose metabolism in obesity	ND	Inhibits BC [67]
FoxC2	Inhibits adipogenesis by inhibiting the induction of PPARγ	ND	Promotes BC [68]
CHOP	Changes the fate of mesenchymal stem cells (MSC) to myocytes and osteocytes rather than adipocytes [69,70]	ND	Invasiveness [71]
Wnt signaling	Inhibits adipogenesis by changing the fate of MSC	Development of mammary gland, its branching and regulating its function [72,73,74,75,76,77]	High grade tumor and poor prognosis [78,79,80]
PREF-1	Important for embryonic WAT and expression of adult adipose tissue [80,81] Maintain preadipocytes state and inhibits adipocyte differentiation [81,82]	Depending on the stimuli from steroid hormones it can differentiate MSC of breast into adipocytes or epithelial cells [83]	High level of PREF-1 inhibits proliferation and invasion, whereas low-level of it is required for these processes [84]
SIRT-1	Inhibits adipogenesis	ND	Controversial role in BC [85,86]
TAZ	Inhibits adipogenesis by repressing PPARγ	Negative regulator of luminal differentiation [87]	Aggressiveness of BC, role in migration, invasion and tumorigenesis [87]

ND = No available data.

## Abbreviations

ECM	Extracellular matrix
BC	Breast cancer
WAT	White adipose tissue
TEBs	Terminal end buds
UCP-1	Uncoupling protein 1
BAT	Brown adipose tissue
PRDM16	PR-domain containing 16
FGF	Fibroblast growth factor
PPAR-γ	Peroxisome proliferator-activated receptor-γ
PGCα	PPAR-γ coactivator α
Cox2	Cyclooxygenase 2
MIR	microRNA
PAT	Pink adipose tissue
WAP	Whey acidic protein
SPP1	Secreted phosphoprotein 1
SFRP1	Secreted Frizzled Related Protein 1
C/EBP	CCAAT-enhancer-binding proteins
ER	Estrogen receptor
EBF	Early B Cell Transcription Factor
SREBP	Sterol regulatory-element binding protein
KLF	Krüppel-like family of transcription factors
EMT	Epithelial to mesenchymal transition
GATA	GATA-binding factor
Fox	Forkhead Box
CTNNB1	Catenin Beta 1
PREF-1	Preadipocyte factor 1
SIRT-1	Sirtuin 1
TAZ	Tafazzin
HER2	Human epidermal growth factor receptor 2
TNBC	Triple-negative BC
LDL	Low density lipoproteins
PAI-1	Plasminogen activator inhibitor 1
FABP4	Fatty acid binding protein 4
IL	Interleukin
TNF	Tumor Necrosis Factor
ADSC	Adipose tissue derived stem cells
OPN	Osteopontin
VEGF	Vascular endothelial growth factor
HGF	Hepatocyte growth factor
NGF	Nerve growth factor
IGF	Insulin growth factor
AIF1	Allograft inflammatory factor 1
EGR2	Early Growth Response 2
AIF1v	AIF1 splice variant
PABC	Pregnancy associated BC
PR	Progesterone receptor
ARLI	Age-related lobular involution
BMI	Body mass index
Aregs	Adipogenesis regulatory cells
APC	Adipocyte precursor cells
PDGF	Platelet-derived growth factor
PDGFRα	Platelet-derived growth factor receptor tyrosine kinase α
NSAIDs	Anti-inflammatory drugs
SVF	Stromal vascular fraction
